# Promising results of a clinical feasibility study: CIRBP as a potential biomarker in pediatric cardiac surgery

**DOI:** 10.3389/fcvm.2024.1247472

**Published:** 2024-02-01

**Authors:** Jana Lücht, Raphael Seiler, Alexa Leona Herre, Liliya Brankova, Raphaela Fritsche-Guenther, Jennifer Kirwan, Dörte Huscher, Hanna Münzfeld, Felix Berger, Joachim Photiadis, Giang Tong, Katharina R. L. Schmitt

**Affiliations:** ^1^ Department of Congenital Heart Disease/Pediatric Cardiology, Deutsches Herzzentrum der Charité – Medical Heart Center of Charité and German Heart Institute Berlin, Berlin, Germany; ^2^ Berlin Institute of Health at Charité – Universitätsmedizin Berlin, Berlin, Germany; ^3^ Metabolomics Platform, Berlin Institute of Health at Charité – Universitätsmedizin Berlin, Berlin, Germany; ^4^ Institute of Biometry and Clinical Epidemiology, Charité – Universitätsmedizin Berlin, Corporate Member of Freie Universität Berlin and Humboldt-Universität zu Berlin, Berlin, Germany; ^5^ Department of Radiology, Charité – Universitätsmedizin Berlin, Corporate Member of Freie Universität Berlin and Humboldt-Universität zu Berlin, Berlin, Germany; ^6^ Department of Congenital Heart Surgery and Pediatric Heart Surgery, Deutsches Herzzentrum der Charité – Medical Heart Center of Charité and German Heart Institute Berlin, Berlin, Germany

**Keywords:** pediatric cardiology, pediatric cardiac surgery, cold inducible RNA-binding protein (CIRBP), inflammation, endothelial dysfunction, biomarker, feasibility study

## Abstract

**Objective:**

*C*old-inducible RNA binding Protein (CIRBP) has been shown to be a potent inflammatory mediator and could serve as a novel biomarker for inflammation. Systemic inflammatory response syndrome (SIRS) and capillary leak syndrome (CLS) are frequent complications after pediatric cardiac surgery increasing morbidity, therefore early diagnosis and therapy is crucial. As CIRBP serum levels have not been analyzed in a pediatric population, we conducted a clinical feasibility establishing a customized magnetic bead panel analyzing CIRBP in pediatric patients undergoing cardiac surgery.

**Methods:**

A prospective hypothesis generating observational clinical study was conducted at the German Heart Center Berlin during a period of 9 months starting in May 2020 (DRKS00020885, https://drks.de/search/de/trial/DRKS00020885). Serum samples were obtained before the cardiac operation, upon arrival at the pediatric intensive care unit, 6 and 24 h after the operation in patients up to 18 years of age with congenital heart disease (CHD). Customized multiplex magnetic bead-based immunoassay panels were developed to analyze CIRBP, Interleukin-1β (IL-1β), Interleukin-6 (IL-6), Interleukin-8 (IL-8), Interleukin-10 (IL-10), Monocyte chemotactic protein 1 (MCP-1), Syndecan-1 (SDC-1), Thrombomodulin (TM), Vascular endothelial growth factor (VEGF-A), Angiopoietin-2 (Ang-2), and Fibroblast growth factor 23 (FGF-23) in 25 µl serum using the Luminex MagPix® system.

**Results:**

19 patients representing a broad range of CHD (10 male patients, median age 2 years, 9 female patients, median age 3 years) were included in the feasibility study. CIRBP was detectable in the whole patient cohort. Relative to individual baseline values, CIRBP concentrations increased 6 h after operation and returned to baseline levels over time. IL-6, IL-8, IL-10, and MCP-1 concentrations were significantly increased after operation and except for MCP-1 concentrations stayed upregulated over time. SDC-1, TM, Ang-2, as well as FGF-23 concentrations were also significantly increased, whereas VEGF-A concentration was significantly decreased after surgery.

**Discussion:**

Using customized magnetic bead panels, we were able to detect CIRBP in a minimal serum volume (25 µl) in all enrolled patients. To our knowledge this is the first clinical study to assess CIRBP serum concentrations in a pediatric population.

## 1 Introduction

Systemic inflammatory response syndrome (SIRS) is a frequent complication after cardiac surgery in children with congenital heart disease (CHD) caused by multiple factors including tissue injury due to surgical incision and the extracorporeal circuit during cardiopulmonary bypass (CPB) (1). During CPB the contact of blood to the synthetic surface of the extracorporeal circuit induces an early phase of systemic inflammation by activation of the complement system and release of proinflammatory cytokines (2). The late response is triggered by ischemia/reperfusion-induced injury leading to endotoxemia due to intestinal barrier changes. This stimulates systemic inflammation even further (2–4), causing and aggravating an impaired vascular endothelial barrier, which can lead to capillary leak syndrome (CLS) (2, 5). Postoperative morbidity is increased as SIRS and CLS are associated with a longer stay on the pediatric intensive care unit (PICU), prolonged mechanical ventilation, and higher demand for catecholamines (5–8).

To date, routinely assessed laboratory markers including C-reactive protein (CRP), Interleukin-6 (IL-6), lactate, and platelet count have been shown to be influenced by cardiac surgery, however they cannot be used to identify patients with SIRS (6). Different diagnostic approaches for CLS have been described including measurement of subcutaneous cytokines and hemoconcentration in infants with hypoplastic left heart syndrome undergoing Norwood stage 1 operation (9, 10). However, as we are currently lacking suitable biomarkers for identifying patients at risk of SIRS and CLS early on, diagnosis and therefore treatment of critically ill patients can be delayed.

CLS is primarily induced by endothelial dysfunction leading to a shift of intravascular fluid and protein to surrounding tissue and cavities, resulting in edema as well as intravasal volume and protein depletion. Vascular barrier and therefore, vascular permeability is controlled by two major components consisting of an inner, lumen facing layer consisting of endothelial glycocalyx and an outer layer, the endothelial cells (11). Furthermore, experimental studies have shown that the endothelial glycocalyx plays an important role in maintaining colloid osmotic gradient and preventing tissue edema (12, 13). Syndecan-1 (SDC-1) is a part of the endothelial glycocalyx that gets released upon glycocalyx degradation and has been described as a marker for glycocalyx shedding (14). Thrombomodulin (TM) is a transmembrane glycoprotein found in the vascular endothelium (15) and serves as a biomarker for endothelial injury as its release as soluble TM is initiated only by endothelial cell injury (16). Both the release of SDC-1 and elevated serum TM has been described in patients with SIRS/sepsis (17–19). Furthermore, Angiopoietin-2 (Ang-2) and vascular endothelial growth factor (VEGF-A) have been described as biomarkers for vascular leakage. Ang-2 is an endothelial growth factor that can be released upon endothelial activation (20). Both Angiopoietin-1 (Ang-1) and Ang-2 bind to the endothelial receptor tyrosine kinase (Tie2) (21). Whereas Ang-1 induces stabilization of the endothelial barrier, Ang-2 has been shown to induce inflammation and vascular leakage (22, 23). VEGF-A has been shown to play an important role in the induction of vascular permeability (24). VEGF-A is produced by neutrophils, macrophages, endothelial and smooth muscle cells, and stored in platelets (25–27). VEGF-A synthesis and release is induced by hypoxia, nitric oxide, coagulation, and bacterial endotoxins (25, 28–30). *In vivo* studies have shown that Ang-2 and VEGF-A are linked in the pathogenesis of vascular leakage as Ang-2 induced vascular barrier changes is synergistically driven by VEGF-A (31, 32). Ang-2 has been described as a biomarker for increased vascular permeability and poor outcome in both adults and children with SIRS and sepsis (33, 34). Additionally, SDC-1, Ang-2, and VEGF-A have also been studied in children after cardiac surgery (35–39).

Cold inducible RNA-binding protein (CIRBP) is a highly conserved 18-kDa nuclear protein belonging to the family of cold-shock proteins (40). It is expressed in various tissues (41) and upregulated upon stimuli including mild to moderate cold stress (28–34°C), ultraviolet radiation, and hypoxia (40, 42, 43). Extracellular CIRBP has been shown to act as a damage associated molecular pattern (DAMP) and has been reported as a novel biomarker for inflammation (44–46). By binding to toll-like receptor 4 (TLR4) and myeloid differentiation factor 2 (MD2) complex (44) as well as triggering receptor expressed on myeloid cells-1 (TREM-1) (47) and Interleukin 6 receptor (IL-6R) (48), CIRBP activates the proinflammatory stress response. *In vitro* and *in vivo* studies have reported CIRBP as a potent mediator of inflammation due to its ability to promote both cytokines and the release of other DAMPs, whereas CIRBP blockage resulted in a significant reduction of inflammation as well as higher survival rates (44, 45).

As both experimental and clinical data have described CIRBP as a mediator of inflammation, it could serve as a potential biomarker for postoperative inflammatory reactions in our pediatric cohort. Furthermore, experimental studies have reported CIRBP to be involved in the pathogenesis of endothelial dysfunction (49). However, to our knowledge CIRBP has not been analyzed in children with CHD after cardiac surgery. Moreover, a potential correlation between sex and age dependency in the amount of CIRBP detectable in serum after cardiac surgery with CHD has not been investigated.

Therefore, we conducted a feasibility study using a customized magnetic bead panel to analyze CIRBP, pro- and anti-inflammatory cytokines, as well as previously described biomarkers for increased vascular permeability at defined time points before and after cardiac surgery.

## 2 Methods

### 2.1 Study design

This prospective observational feasibility study was conducted at the German Heart Center Berlin after approval by the Ethics Committee of Charité—Universitätsmedizin Berlin, Germany (decision EA2/180/19). The study was registered with the German register for clinical studies before patients' recruitment (registration number: DRKS00020885; https://drks.de/search/de/trial/DRKS00020885). Written consent was obtained from the parents of each patient before study inclusion. Patients younger than 18 years receiving a cardiac surgery at our center were enrolled. Exclusion criteria were as follows: gestational age ≤ 37 weeks, a known maternal alcohol or substance abuse during pregnancy, immunodeficiencies or immunosuppressive medication, syndromic diseases (e.g., Trisomy 21 and 18), and congenital kidney disease.

#### 2.1.1 Preoperative, operative and postoperative management

19 Patients were enrolled over a period of 9 months starting in May 2020. Anesthesia was performed by standardized protocols using propofol (3–5 mg/kg), rocuronium (1 mg/kg) and sufentanil (1 µg/kg) for induction and propofol (5 mg/kg/h), remifentanil (1–3 µg/kg/min), and dexmedetomidine (0.25–1 µg/kg/h) for maintenance. All patients received a urinary catheter, a central venous line as well as a femoral or radial arterial cannula upon induction of anesthesia. Most patients received a single shot dexamethasone (0.15 −0.5 mg/kg) before cardiac surgery. Core temperature was measured during surgery and postoperatively on the intensive care unit via a rectal temperature probe.

Cardiac surgery was performed depending on the underlying anomaly. On-pump beating heart surgery was performed in 5 patients (26%) to prevent reperfusion injury, aortic cross-clamping was necessary in 14 patients (74%). CPB was performed using polyvinyl tubing, roller pumping (mast-mounted pump, Stöckert Instruments, München, Germany), and a hollow fiber membrane oxygenator (Capiox RX05, Terumo Corp., Tokyo, Japan). Priming of the extracorporeal circuit was achieved using a balanced electrolyte solution (Ionosteril, Fresenius Kabi, Bad Homburg, Germany), and heparin (500 IE/kg), as well as tranexamic acid (10–15 mg/kg at initiation of CPB and 1–3 mg/kg/h for maintenance) was administered. Flowrate was maintained at 3 L/m^2^ body surface area during cardiopulmonary bypass. Upon weaning from cardiopulmonary bypass, protamine sulfate was administered according to remaining heparin effect (usually 10 mg/1,000 IE). After the operation, patients were transferred to our intensive care unit. For postoperative management of analgesia sufentanil (0.2–0.6 µg/kg/h) or morphine (30–60 µg/kg/h) or piritramide (0.1 mg/kg) plus additive metamizole (10 mg/kg) and paracetamol (10 mg/kg) were standardly administered. Dexmedetomidine (0.25–1 µg/kg/h) was given for postoperative adjuvant sedation.

#### 2.1.2 Protocol of blood sample acquisition

**Figure 1:**
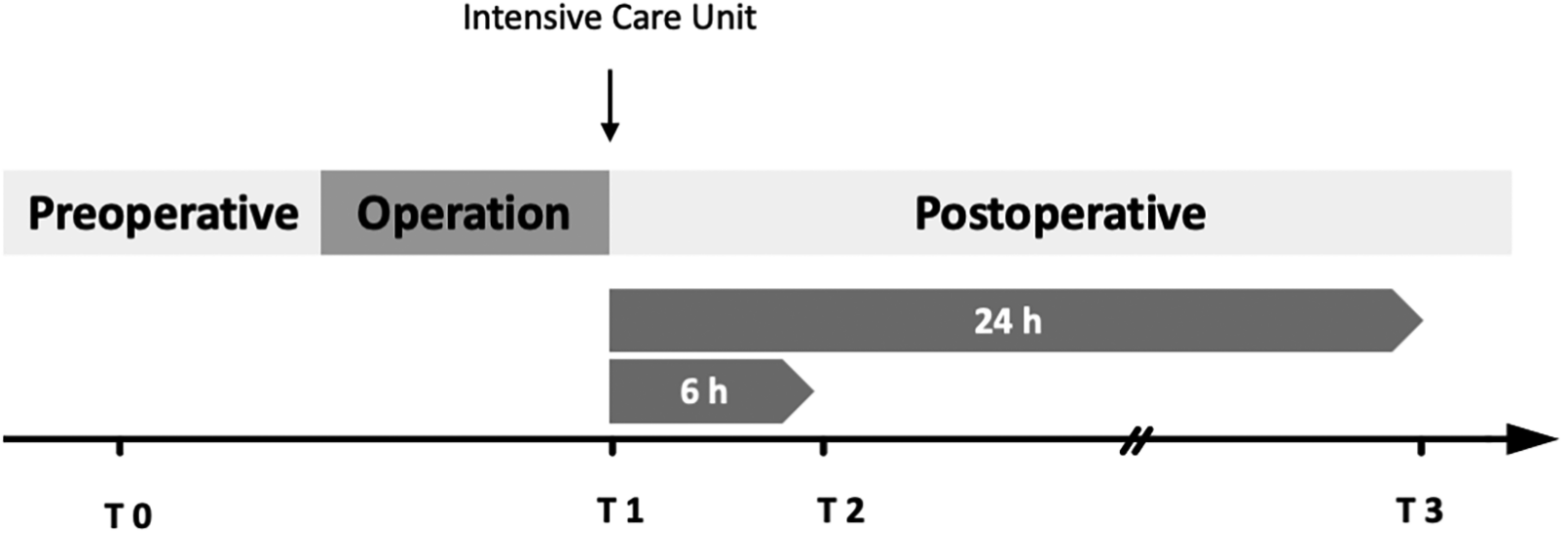
Protocol of time points for blood sample collection. T0 = Baseline, collection after induction of anesthesia via central venous line; T1 = collection upon arrival on the intensive care unit; T2 = 6 h after T1; T3 = 24 h after T1. Blood samples were obtained in Serum-Gel Microvette® 500, centrifuged at 26 × g for 10 min and stored at −80°C until analysis.

Blood samples for biomarker analysis were obtained via the central venous line preoperatively after the induction of anesthesia (T0) as well as postoperatively upon arrival on the pediatric intensive care unit (PICU) (T1), and both 6 and 24 h after the operation (T2 and T3 respectively, Figure 1). We collected 1 ml blood for patients ≤15 kg and 2 ml of blood for patients >15 kg at each timepoint in Serum-Gel Microvette® 500 (20.1344 Sarstedt, Nümbrecht, Germany). Samples were centrifuged at 26 × g for 10 min and frozen temporarily at −8°C until final storage at −80°C.

#### 2.1.3 Study outcomes

The aim of this clinical feasibility study was to measure perioperative CIRBP serum levels as well as concentrations of cytokines and biomarkers for endothelial dysfunction in a very small sample volume (25 µl/sample) from children undergoing cardiac surgery. Clinical primary endpoints were defined as duration of mechanical ventilation, duration of the intensive care unit stay, as well as the level of inotropic support. Administered catecholamines were documented hourly from the time of T1 (arrival on PICU) up to 72 h after operation and both maximum and mean vasoactive inotropic scores were calculated according to *Gaies* et al. (50).

Secondary endpoints were defined as 30-day mortality, as well as postoperative complications such as infection, arrhythmia, acute kidney injury, and signs for capillary leakage. Acute kidney injury was defined according to the KDIGO guidelines (51). Serum creatinine was assessed preoperatively in a standardized preoperative blood collection (usually 1 day before the operation, including analysis of red and white blood cell count, platelet count, CRP, liver and kidney values, as well as blood clotting analysis) at the studied time points (Figure 1 Protocol of time points) up to 96 h after operation, as well as maximum creatinine value during the hospital stay. For assessment of CLS, we analyzed both positive fluid overload exceeding >10% bodyweight during the first 72 h after operation and a subcutaneous-thoracic ratio (S/T) with a threshold of >12.6% calculated from a chest x-ray based on *Sonntag* et al.*,* 24 to 72 h after operation (52). We utilized both tools as capillary leak syndrome is a frequently described complication after pediatric cardiac surgery, but we are currently lacking objective diagnostic criteria for this postoperative complication. Chest x-rays are part of postoperative diagnostics especially after extubation but are not performed routinely especially in pediatric patients. In our experience capillary leak syndrome is a clinical diagnosis. As fluid balance was assessed and analyzed postoperatively, we included this analysis. Chest-x-rays were analyzed by an experienced radiologist. x-rays were not included in the analysis if soft tissue was cut off. Fluid balance was documented for the first 72 h after operation (including the operation period) and fluid overload (FO) was calculated using the fluid balance method as follows (53):

$$\text{\%}\mathrm{FO}=\;\frac{\mathrm{Total}\;\mathrm{fluid}\;\mathrm{intake}\;(\mathrm{ml})-\mathrm{Total}\;\mathrm{fluid}\;\mathrm{outtake}\;(\mathrm{ml})}{\mathrm{pre}-\mathrm{operative}\;\mathrm{weight}\;(\mathrm{kg})}\times \;100$$

Furthermore, the amount of blood products (erythrocytes, thrombocytes, fresh frozen plasma), clinical and laboratory chemical signs for infection and organ dysfunction (body temperature, CRP, leukocytes, urea, creatinine, aspartate transferase (AST), alanine transaminase (ALT), creatinine kinase (CK), creatinine kinase-myoglobin binding (CK-MB), lactate, and lactate dehydrogenase (LDH) and microbiological cultures were assessed.

For risk adjustment concerning in-hospital mortality we used the Risk Adjustment for Congenital Heart Surgery 1 (RACHS-1) consensus-based scoring system (54).

### 2.2 Magpix® bead-based analysis

Proteins were measured via MagPix® analysis (Merck/Millipore) using customized magnetic bead panels (Merck KGaA, Darmstadt, Germany) as listed in Table 1. Serum samples were defrosted and diluted according to manufactureŕs recommendations. Briefly, for SPRCUS1273 neat samples were used, whereas samples were diluted at 1:8 for SPRCUS1355. For data acquisition, Xponent (Merck KGaA, Darmstadt, Germany, version 4.2) and Milliplex (Merck KGaA, Darmstadt, Germany, version 5.1.0.0) software were used. All samples showed a bead count of >100. Serum background was subtracted from the mean fluorescent intensities (MFI) and concentration (pg/mL) was determined using an 8-point calibration curve including matrix (best fit from Milliplex Analyst software was used). Single samples were measured. For determining the instrumental variance, pooled quality control (QC) samples were used as quality control (*n* = 3 analysed at the beginning, middle and end of the run). The biological variability was higher compared to the technical variability. One patient's sample at T3 was clotted in the well, consequently the assay SPRCUS1273 could not be run for this sample.

**Table 1:** List of analyzed serum biomarkers and assay IDs.

Protein	Assay ID
CIRBP	SPRCUS1273
IL-1β	SPRCUS1273
IL-6	SPRCUS1273
IL-8	SPRCUS1273
IL-10	SPRCUS1273
MCP-1	SPRCUS1273
FGF-23	SPRCUS1273
VEGF-A	SPRCUS1273
Ang-2	SPRCUS1355
SDC-1	SPRCUS1355
TM	SPRCUS1355

### 2.3 Statistical analysis

Numbers are presented as counts and percentages for categorical data and mean with standard deviation or median with range for continuous data. Data and respective tests were considered as a non-confirmatory, hypothesis-generating pilot study. The Wilcoxon test was used to compare post-surgery measurements with the pre-surgery biomarker value. No replacement of missing biomarker values was applied for analysis. *P*-values of <0.05 were considered significant; adjustment for 3 parallel tests of the 3 post-surgery measurements compared to the pre-surgery measurement, in case of gender comparisons for the 4 parallel tested time points per biomarker was done with Bonferroni correction. Longitudinal analysis of biomarker data was conducted with linear mixed-effects models (R packages lmer4 and lmerTest) with splines of grade 3 for time including age, sex, and duration of surgery as covariables. Considering the complexity of the linear mixed-effects models regarding the available case numbers, effects with *p*-values < 0.1 were also considered as “significant” signals. IBM SPSS Statistics 28.0 and R version 4.0.2 was used for analysis.

## 3 Results

For this pilot study, we enrolled 19 patients consisting of 10 males and 9 females. The median age was 2.9 years ranging from 0 to 18 years. Our cohort did not include neonatal patients. Demographic and data on diagnoses and procedures are summarized in Table 2. The study cohort represented a broad range of patients with congenital heart disease concerning both age and complexity of anomalies treated at our center. All patients survived until hospital discharge. On-pump beating cardiac surgery was performed in 5 patients (2 pulmonary valve replacements, 2 Glenn-anastomosis, and 1 modified-Fontan procedure). Aortic cross-clamping was necessary in 14 patients. In 6 patients systemic mild-moderate hypothermia (32–35.9°C) and in 5 patients moderate deep hypothermia (<30°C–31.9°C, with the lowest temperature 28°C) was applied, whereas 8 patients were kept at normothermia during CPB.

**Table 2:** Demographic and clinical data.

	Sex	
	Male	Female
Number of patients: 19	10 (53%)	9 (47%)
Demographic data		
Median age (years), (range)	2 (0–15)	3 (0–18)
0–12 months	4 (40%)	3 (33%)
13–24 months	1 (10%)	
25 months – 12 years	3 (30%)	5 (56%)
>12 years	2 (20%)	1 (11%)
Diagnoses (number)		
* *	AS (1)	AI (1)
* *	ASD (1)	ASD (1)
* *	AVSD (2)	ccTGA, MA, PA (1)
* *	Ebstein's anomaly (1)	DORV, TGA, PS, VSD (1)
* *	CoA, AS, VSD (1)	HLHS (1)
* *	PA, VSD (1)	PAPVD (1)
* *	TA (1)	TOF (1)
* *	VSD (2)	TA (1)
* *		VSD, ASD (1)
Procedures (number)		
* *	Complex surgery (2)	Complex surgery (1)
* *	ASD closure (1)	Aortic valve replacement (1)
* *	Modified Fontan operation (1)	ASD closure (1)
* *	AVSD correction (1)	Modified Fontan operation (1)
* *	Pulmonary replacement (2)	Glenn Shunt (2)
* *	Ross operation (1)	PAPVD correction (1)
* *	VSD closure (2)	TOF repair (1)
* *		VSD closure (1)
Characteristics of operation and CPB (median; range)		
RACHS-1	2 (1–6)	2 (1–4)
Operation time (min)	343 (135–777)	331 (177–754)
CPB-time (min)	180 (60–480)	180 (60–480)
Aortic cross-clamp time (min)	45 (0–259)	67 (0–266)
Perfusion time (min)	133 (60–467)	162 (56–480)
Re-perfusion time (min)	22 (0–184)	13 (0–178)

AS, Aortic stenosis; AI, Aortic insufficiency; ASD, Atrial septal defect; AVSD, Atrioventricular septal defect; CoA, Coarctation; (cc)TGA,(congenital corrected) transposition of the great arteries; DORV, Double outlet right ventricle; HLHS, Hypoplastic left heart syndrome; MA, Mitral atresia; PA, Pulmonary atresia; PAPVD, Partial anomalous pulmonary venous connection; PS, Pulmonary stenosis; TA, Tricuspid atresia; TOF, Tetralogy of Fallot; VSD, Ventricuklar septal defect; RACHS-1, Risk Adjustment for Congenital Heart Surgery 1; CPB, cardiopulmonary bypass.

**Figure 2:**
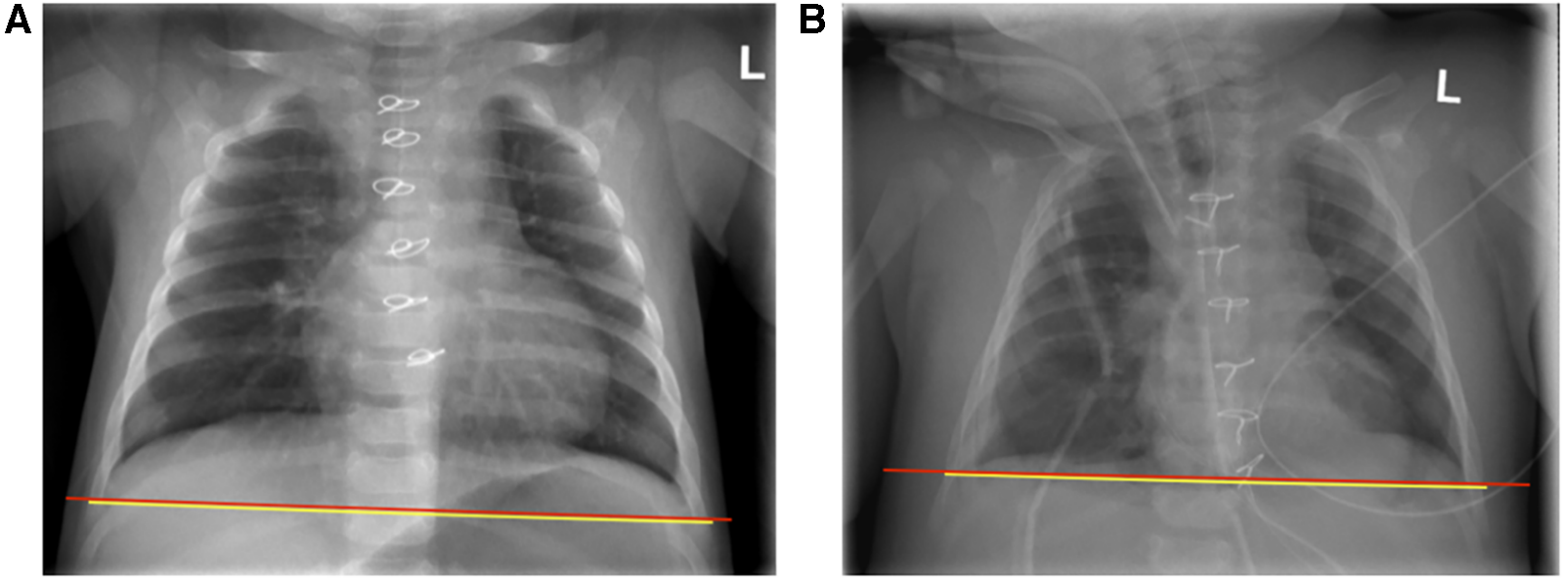
Example of chest x-ray analysis for assessment of subcutaneous-thoracic (S/T) ratio according to *sonntag* et al. (52) (**A**) Pre- and (**B**) postoperative x-rays of a patient with an S/T-ratio 16.8.

Duration of mechanical ventilation and maximum as well as mean vasoactive-inotropic score (VIS) was comparable in both male and female patients. However, duration of stay on the PICU was longer in male compared to female patients (median 31 h and 9 h, respectively as summarized in Table 3). Acute kidney injury as defined by KDIGO criteria, infections, and arrhythmia presented frequent complications in our patient cohort (Table 3) (51). One patient showed respiratory failure, hemodynamic instability, and increased demand for volume and catecholamines after modified Fontan procedure and was re-intubated during the first 24 h after initial surgery (55, 56). After catheterization on the first postoperative day, the patient received a re-thoracotomy and takedown of the Fontan-procedure. Another patient showed an atrioventricular block III° postoperatively and received a transvenous pacemaker 10 days after initial cardiac surgery. S/T-ratio and fluid overload were assessed as criteria for CLS. In 7 patients S/T ratio was not analyzable due to a cut off soft tissue on the x-ray. 3 patients showed an S/T-ratio >12.6% during the first 72 h after CPB (an exemplary chest x-ray analysis is shown in Figure 2). During the first 24 h after operation both male and female patients presented a median fluid overload of 16% (Table 3). None of the enrolled patients died during hospitalization, 30-day mortality was 0% in both male and female patients. One patient with univentricular heart physiology was released after initial shunt operation and 6 weeks later readmitted to another hospital due to gastroenteritis. Two days after readmission the patient had an in-hospital cardiac arrest most likely due to shunt thrombosis, was transferred to our hospital under resuscitation conditions, where we ended the treatment due to the poor prognosis.

**Table 3:** Perioperative characteristics.

	Sex	
	Male	Female
Number of patients: 19	10 (53%)	9 (47%)
Mechanical ventilation (h)	9.7 (5.5–270)	7.6 (4.9–339)
Length of PICU stay (h)	30.8 (7.2–463)	8.75 (7.2–890)
VIS mean (median; range)		
1–24 h	0.68 (0.01–13.02)	0.30 (0–7.2)
25–48 h	0 (0–6.7)	0 (0–4.57)
49–72 h	0 (0–5.1)	0 (0–1.97)
1–72 h	0.33 (0–7.6)	0.19 (0–4.61)
VIS max (median; range)		
1–24 h	3.3 (0.2–26.02)	3.46 (0–12.89)
25–48 h	0 (0–11.8)	0 (0–6.29)
49–72 h	0 (0–16.4)	0 (0–2.91)
1–72 h	3.33 (0.2–26.02)	3.46 (0–12.89)
Complications (number)		
Infection	1	3
Arrhythmia	2	0
Acute kidney injury	3 (30%)	2 (22%)
Postoperative S/T-ratio >12.6% up to 72 h (range in %)	1 (14.1)	2 (13.7–16.8)
Fluid overload > 10% (range)		
24 h	6 (2–16)	4 (−1–16)
48 h	0 (−9 - 4)	0 (−7 - 3)
72 h	0 (−7 - 0)	0 (−6 - 3)
Cardiac arrest	0	0

PICU, Pediatric intensive care unit; VIS, Vasoactive-inotropic Score; Max, maximum; S/T-ratio, Subcutaneous-thoracic ratio.

### 3.1 Analysis of biomarkers

We collected blood and urine samples at the respective timepoints for all patients (Figure 1. Protocol of timepoints). The study protocol included blood and urine sample collection at the same timepoints. The urine samples were used to discover new urinary biomarker for early detection of acute kidney injury using proteomics analysis. However, not in all 19 patients included in the feasibility study urine samples could be collected during all timepoints. Furthermore, as this manuscript focuses on the feasibility of the customized magnetic bead panels, data on proteomic analysis will be published separately. For 15 patients, blood samples were obtained at all timepoints. In 3 patients T3 was not obtained as patient care on PICU did not allow sample acquisition at that timepoint, and one patient needed a re-thoracotomy at this timepoint.

Our first objective—the reliable measurement of protein concentrations in the serum of children in the clinical setting—was achieved. In short, the analyzed biomarkers could be detected using the customized magnetic bead panels. For IL-6, IL-8, MCP1, and FGF-23 a value above the limit of detection/quantification (LOD/Q) could be quantified in all samples. For CIRBP *n* = 9 samples showed values below the LOD/Q, while for IL-1β *n* = 10 sample values were below the LOD/Q. For IL-10 and VEGF-A one sample each was below LOD/Q.

#### 3.1.1 Cytokines analyzed

**Figure 3:**
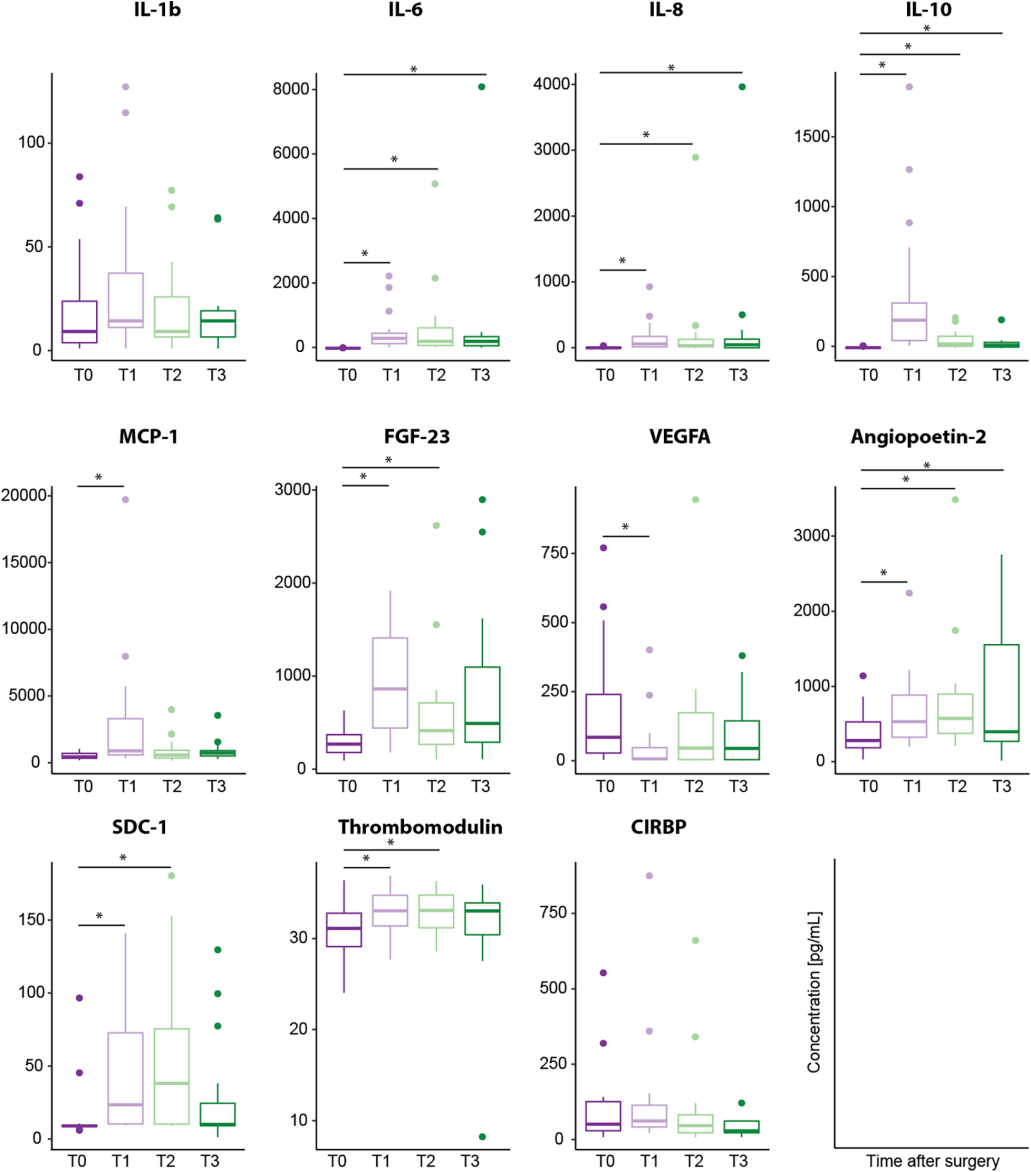
Biomarker serum concentration [pg/ml] analyzed using customized magnetic bead panels. Timepoints of blood sample collection: T0 = baseline, at anesthesia induction; T1 = arrival on the intensive care unit; T2 = 6 h after T1; T3 = 24 h after T1. Dots represent statistical outliers. Statistical analysis was performed using the Wilcoxon test; *p*-values were adjusted for 3 parallel tests (all post-operative measurements vs. baseline) and *p < 0.05 was considered statistically significant.

**Figure 4:**
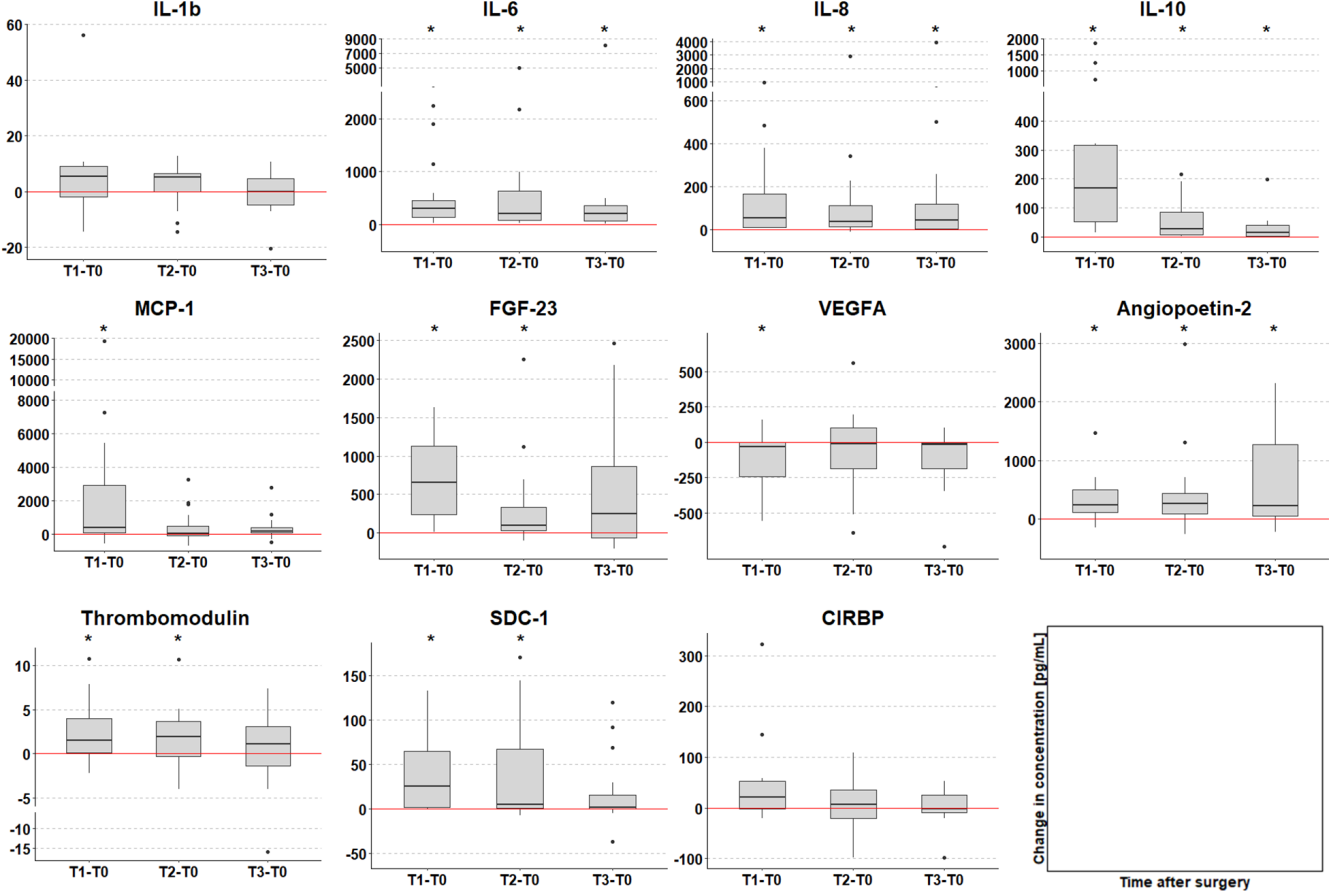
Change in biomarker serum concentration [pg/ml] compared to T0 (time of anesthesia induction). Timepoints of post-surgery blood sample collection: T1 = arrival on the intensive care unit (median 8 h after T0); T2 = 6 h after T1; T3 = 24 h after T1. The red line at 0 indicates no change, positive values increase and negative values decrease compared to T0. Dots represent statistical outliers. *p*-values were adjusted for 3 parallel tests; stars indicate significant changes (*p* < 0.05).

IL-6, IL-8, MCP-1, and IL-10 all showed low baseline serum concentrations at induction of anesthesia (T0; Figure 3). In comparison to baseline values, both pro-inflammatory cytokines IL-6 and IL-8 were significantly increased at all postoperative timepoints (T1, IL-6 *p* < 0.001, IL-8 *p* < 0.001; T2, IL-6 *p* < 0.001, IL-8 *p* < 0.001; T3, IL-6 *p* = 0.002, IL-8 *p* = 0.002). Furthermore, IL-10 serum concentration was significantly increased at all postoperative timepoints (T1, *p* < 0.001; T2, *p* < 0.001; T3, *p* = 0.007). MCP-1 serum concentration was significantly higher directly after surgery (T1, *p* = 0.025,). Individual differences between baseline and the three postoperative time points are displayed in Figure 4, for all cytokines analyzed.

IL-1β baseline levels were generally higher than other analyzed cytokines, and serum concentrations showed a non-significant increase directly after surgery (T1). Overall, no significant increase in IL-1β serum concentration was observed (Figures 3, 4).

**Figure 5:**
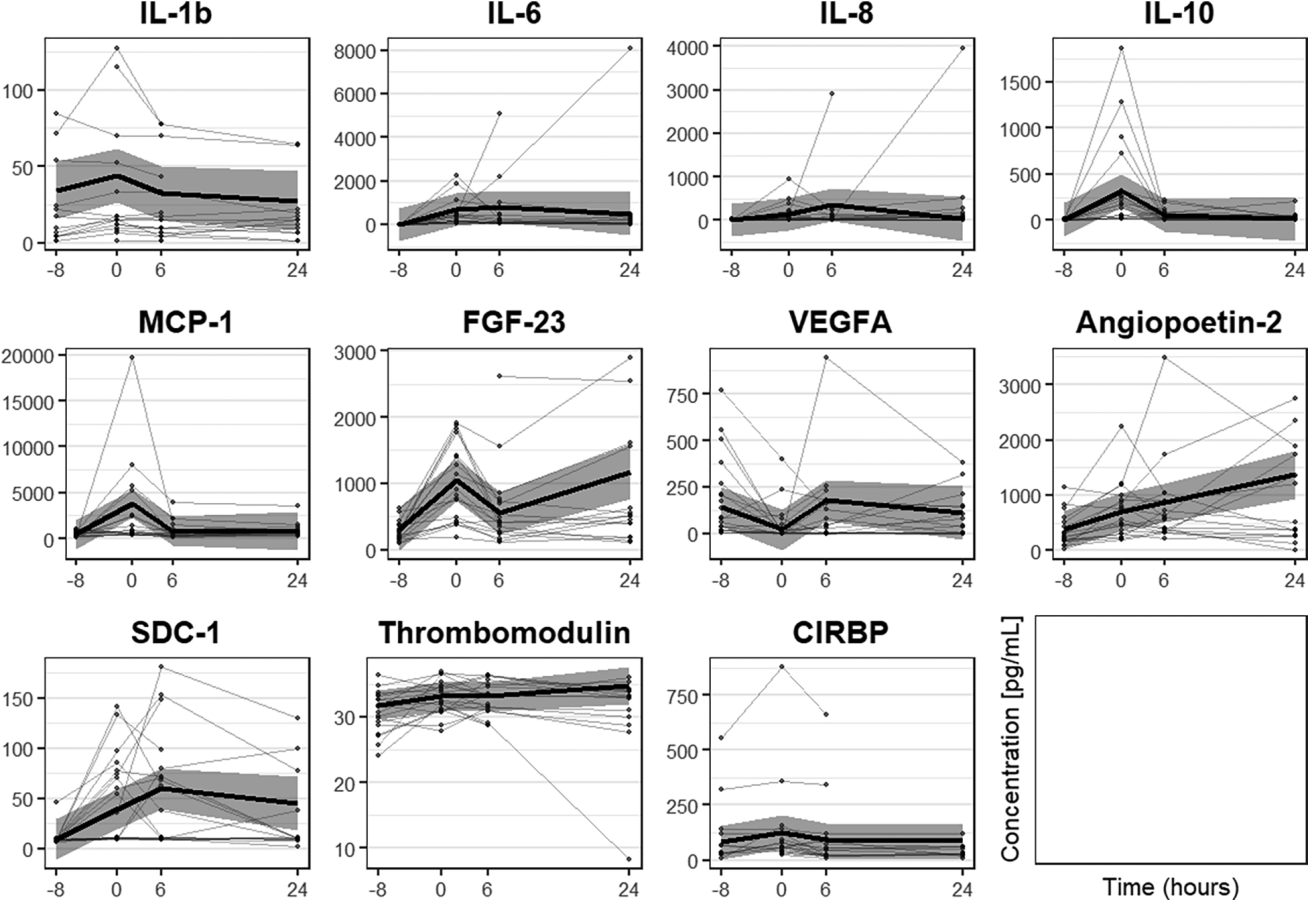
Course of biomarker serum concentration [pg/ml] analyzed with linear mixed-effects models adjusted for age, sex and duration of surgery. The model estimation is shown as bold black line with 95% confidence interval. Dots with connecting lines represent individual courses of biomarker serum concentrations for single patients. Timepoints of blood sample collection: T0 = at anesthesia induction (median 8 h before T1); T1 = arrival on the intensive care unit (=0 h); T2 = 6 h after T1; T3 = 24 h after T1.

**Figure 6:**
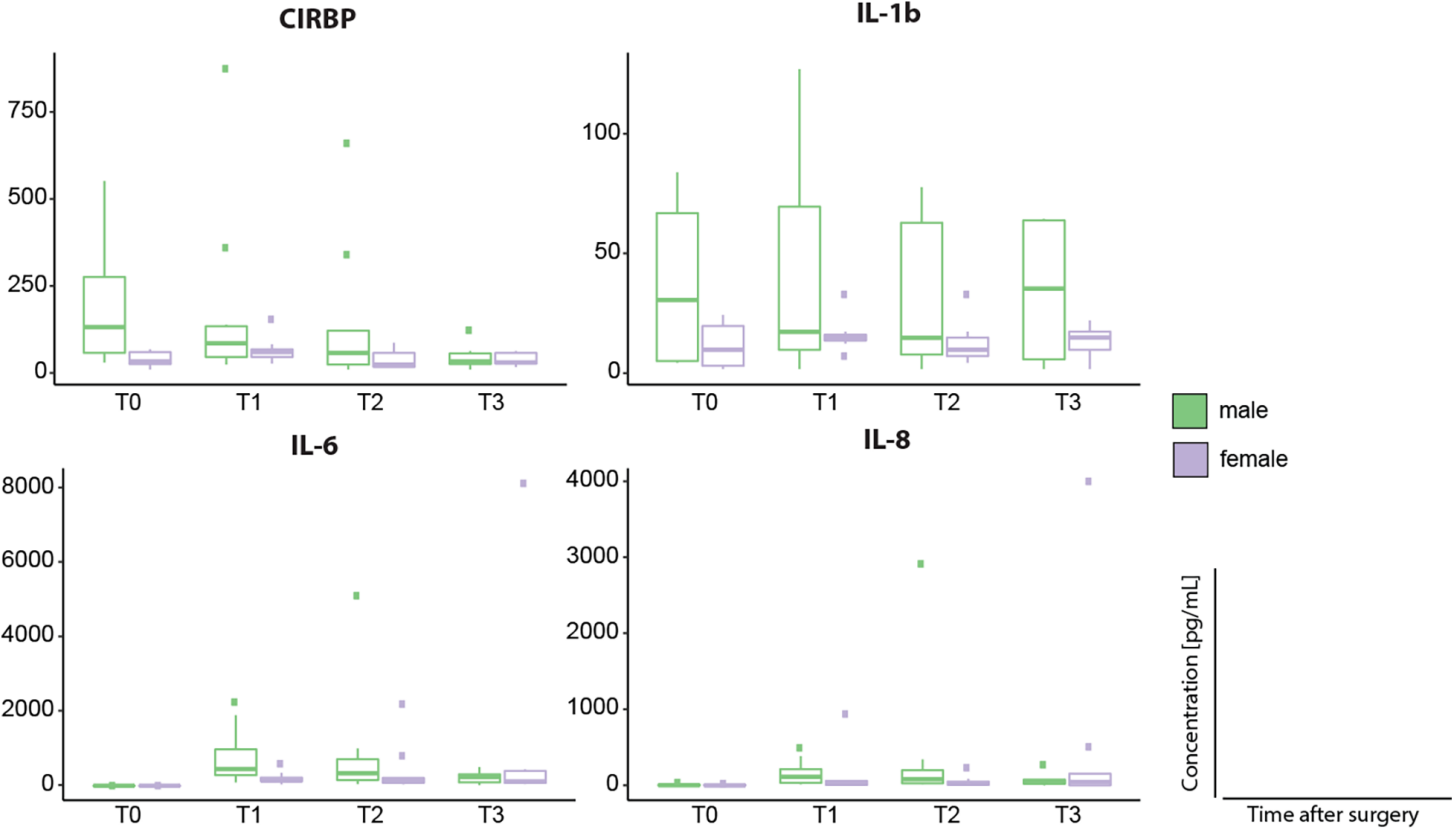
Sex associated differences of CIRBP, IL-1β, IL-6 and IL-8 serum concentrations [pg/ml]. Timepoints of blood sample collection: T0 = baseline, at anesthesia induction; T1 = arrival on the intensive care unit; T2 = 6 h after T1; T3 = 24 h after T1. Dots represent statistical outliers.Statistical analysis was performed using the Mann-Whitney test; *p*-values were adjusted for 4 parallel tests for each biomarker; none of the differences, also due to small case numbers, were statistically significant.

Furthermore, we examined the course of serum biomarkers concentrations with linear mixed-effects models adjusted for age, sex, and duration of surgery (Figure 5). IL-1β, IL-10, and MCP-1 serum concentrations increased directly after surgery and decreased at 6 and 24 h after operation (T2 and T3) upon returning to baseline levels. Whereas IL-6 showed a persistent increased concentration directly after operation, IL-8 peaked at T2 (6 h after operation) and returned to baseline level over time. Furthermore, linear mixed-effect models for the course of IL-1β serum concentration revealed the interaction of female sex and time as potentially significant (β(SE) = 14.3(7.7), *p* = 0.071; Table 4), indicating a more pronounced time course for female sex. Interestingly, we observed a tendency in increased IL-1β serum concentrations at all analyzed timepoints in male compared to female patients, however, this difference was not statistically significant (Figure 6).

**Table 4:** Estimates of the linear mixed-effects models for the course of biomarkers over the 4 time points of measurement adjusted for age, sex and duration of surgery, and respective interaction terms with time. For significant parameters the *p*-value is shown in bold fond. Timepoints of blood sample collection in the model were: −8 h (T0) = at anesthesia induction; 0 h (T1) = arrival on the intensive care unit; 6 h (T2) = 6 h after T1; 24 h (T3) = 24 h after T1.

	IL1b		IL6		IL8		IL10		MCP1		FGF23	
	ß (SE)	*p*	ß (SE)	*p*	ß (SE)	*P*	ß (SE)	*p*	ß (SE)	*p*	ß (SE)	*p*
(Intercept)	37.1 (18.2)	0.054	3.3 (708.1)	0.996	11.9 (365.6)	0.974	15.9 (182)	0.931	558.5 (1,522.7)	0.715	319.8 (313.0)	0.313
Age	1.3 (1.3)	0.334	−0.1 (52.4)	0.999	0.0 (27.1)	0.999	−0.4 (12.7)	0.974	−23.9 (112.7)	0.833	−3 (23.2)	0.897
Time	−23.5 (20.5)	0.262	1,137 (2,146.5)	0.599	482.6 (1,155.3)	0.678	−829.7 (524.1)	0.121	−5,159 (4,630)	0.271	−1,516.5 (798.2)	0.065
Time²	−18.9 (18.2)	0.306	−111.8 (1,589.2)	0.944	88.5 (857.7)	0.918	338.6 (409.3)	0.413	1,604.6 (3,428.6)	0.642	−288.9 (587.6)	0.626
Time³	−14.8 (11.3)	0.202	−120.2 (1,025.3)	0.907	411.9 (548.2)	0.456	−335.4 (250.9)	0.188	−2,270.8 (2,210.7)	0.310	−769.5 (386.4)	0.053
Female sex	−23.1 (13.5)	0.103	−1.0 (539.9)	0.998	−1.6 (278.8)	0.995	−5.5 (133)	0.967	69 (1,161)	0.953	−56.7 (238.6)	0.813
Duration of surgery	−1.6 (2.4)	0.531	0.0 (98.9)	1.000	0.1 (51.1)	0.998	−0.7 (24.1)	0.978	5.5 (212.8)	0.979	1.8 (43.7)	0.967
Age *time	−0.9 (1.3)	0.476	14.4 (152)	0.925	−35.9 (82)	0.663	15.8 (37.1)	0.674	311.3 (327.9)	0.348	1.8 (56.2)	0.974
Age * time²	0.8 (1.2)	0.489	−102.9 (115.2)	0.377	−33.4 (62.2)	0.594	−6.6 (28.4)	0.816	−216.5 (248.5)	0.389	−23.6 (42.5)	0.583
Age * time³	−0.8 (0.6)	0.221	−65.2 (70.2)	0.358	6.3 (37.8)	0.868	4.1 (17.1)	0.812	136.2 (151.4)	0.373	−2.5 (26.1)	0.924
Female sex * time	18.0 (14.2)	0.212	−318.5 (1,578.9)	0.841	−865.1 (851.6)	0.314	−243.4 (385.5)	0.531	3,240.2 (3,406.3)	0.347	186.2 (584.7)	0.752
Female sex * time²	2.6 (13)	0.844	−254.6 (1,203)	0.833	138.1 (648.6)	0.832	133.9 (299.9)	0.658	−1,380.4 (2,595.3)	0.598	−137.7 (445.9)	0.759
Female sex * time³	14.3 (7.7)	0.071	744.6 (784.5)	0.348	392.6 (422)	0.356	−76.2 (191.7)	0.693	1,451.1 (1,692.2)	0.396	−431.1 (291.8)	0.147
Duration of surgery * time	1.2 (2.9)	0.679	−103.4 (313.4)	0.743	44.2 (168.3)	0.794	49.6 (76.4)	0.519	−274.1 (676)	0.687	110 (117.1)	0.353
Duration of surgery * time²	3.0 (2.1)	0.173	322.7 (229.3)	0.167	67.7 (123.7)	0.587	−2.3 (56.6)	0.967	440.3 (494.7)	0.378	274.4 (84.8)	0.002
Duration of surgery * time³	0.7 (1.6)	0.685	92.1 (167.2)	0.584	−74.6 (88.2)	0.401	15.3 (40.5)	0.707	−92.7 (360.2)	0.798	187.6 (64.7)	0.006

cont.	VEGFA		Ang-2		TM		SDC1		CIRBP	
	ß (SE)	*p*	ß (SE)	*p*	ß (SE)	*p*	ß (SE)	*p*	ß (SE)	
(Intercept)	168.7 (105.5)	0.116	195.7 (330.1)	0.557	74.6 (103.6)	0.481	10.3 (19.6)	0.602	33.2 (2.1)	**<0** **.** **001**
Age	−16.2 (7.8)	**0** **.** **043**	−20.4 (24.4)	0.409	−3.6 (8.1)	0.660	−0.3 (1.5)	0.819	−0.3 (0.2)	0.093
Time	686.5 (297.7)	**0** **.** **026**	−364.4 (775.7)	0.641	−117.5 (82)	0.163	−34.3 (52.6)	0.518	−0.8 (5.2)	0.882
Time²	−52.8 (219.7)	0.811	152.7 (597.4)	0.800	−63.2 (69.3)	0.369	−45.3 (40.5)	0.270	1.2 (4.0)	0.772
Time³	272.8 (143.2)	0.063	−365.6 (376.5)	0.337	−58.2 (48.1)	0.236	−28 (25.4)	0.277	−3.5 (2.5)	0.177
Female sex	86.8 (80.4)	0.286	−67 (251.7)	0.791	91.5 (80.3)	0.270	3.3 (15.0)	0.825	−2.1 (1.6)	0.197
Duration of surgery	8.6 (14.7)	0.562	47.8 (46.1)	0.307	−4.0 (14.4)	0.784	0.1 (2.7)	0.970	0 (0.3)	0.924
Age *time	6.3 (21.3)	0.770	112.5 (57.4)	0.057	2.3 (6.1)	0.703	7.1 (3.9)	0.074	0.3 (0.4)	0.386
Age * time²	17.9 (16)	0.270	11.7 (44.0)	0.791	0.3 (7.2)	0.966	1.7 (3.0)	0.580	0.2 (0.3)	0.524
Age * time³	3.6 (9.9)	0.720	−45.4 (26.6)	0.096	2.6 (3.4)	0.457	5.8 (1.8)	**0** **.** **002**	−0.2 (0.2)	0.272
Female sex * time	−448.5 (223.4)	0.051	−296.4 (591.5)	0.619	−20.9 (63.8)	0.746	−57 (40.1)	0.163	0.5 (4.0)	0.906
Female sex * time²	−182.5 (168)	0.283	−279.5 (458.1)	0.545	22.3 (60.7)	0.716	−8.1 (31.0)	0.795	0.2 (3.1)	0.955
Female sex * time³	−158.8 (110.5)	0.158	−498.5 (300.4)	0.104	−10.8 (34.5)	0.756	−36.4 (20.2)	0.079	−4.9 (2.0)	**0** **.** **020**
Duration of surgery * time	−68.7 (43.7)	0.123	46.3 (108.4)	0.672	9.2 (12.7)	0.474	10.1 (7.4)	0.177	−0.1 (0.7)	0.921
Duration of surgery * time²	−22.3 (31.7)	0.485	145.5 (85.2)	0.095	16.5 (9.4)	0.089	18.4 (5.8)	**0** **.** **003**	0.2 (0.6)	0.673
Duration of surgery * time³	−36.7 (23.8)	0.129	231.7 (60.7)	**<0** **.** **001**	3.8 (7.6)	0.619	3.8 (4.1)	0.351	1.1 (0.4)	**0** **.** **011**

#### 3.1.2 Biomarkers for endothelial dysfunction

Serum concentrations of SDC-1, Ang-2, and TM increased significantly after operation. SDC-1 serum concentration was low at induction of anesthesia and showed a significant increase both directly (T1, *p* < 0.001) and 6 h after surgery (T2, *p* = 0.002) in comparison to baseline (T0) (Figures 3A,B). Furthermore, TM serum levels increased directly after surgery (T1, *p* = 0.042) and 6 h (T2, *p* = 0.047) compared to baseline values (Figures 3, 4) with serum concentrations undulating around 30 pg/ml (Figure 3A). Ang-2 serum concentrations increased significantly at all postoperative timepoints (T1, *p* = 0.002; T2, *p* = 0.004; T3, *p* = 0.016, Figures 3, 4).

VEGFA showed a different dynamic with a significant decrease in serum levels directly after surgery (T1, *p* = 0.039) in comparison to baseline (Figures 3, 4).

Analyzing the course of biomarker serum concentrations, SDC-1 increases postoperatively, showing a peak at 6 h after operation, whereas both TM and Ang-2 serum concentration increase over time (Figure 5). Furthermore, modelling the course of SDC-1 and TM serum concentrations, we found age and/or the interaction of time with age, female sex, or duration of surgery to show statistical significance (all *p* < 0.1, Table 4). For TM serum concentrations indicating stronger fluctuation with higher age and increasing duration of surgery flattened by female sex. For SDC-1 suggesting a generally lower level with increasing age and stronger fluctuation with increasing duration of surgery flattened by female sex. The course of Ang-2 serum concentration also seems to be influenced by age and duration of surgery but not by female sex (Table 4).

#### 3.1.3 Fibroblast growth factor (FGF-23)

Fibroblast growth factor FGF-23 has been previously described as a biomarker for acute kidney injury (AKI) after pediatric cardiac surgery (57–60). As we plan to analyze potential biomarkers for acute kidney injury in our clinical trial, we included FGF-23 in our customized magnetic bead panel. FGF-23 concentration significantly increased directly after operation as compared to preoperative serum levels (T1, *p* < 0.001), and remained significantly higher until 6 h after operation (T2 *p* = 0.007, Figures 3, 4).

Analyzing the course of serum concentration with linear mixed-effect models, FGF-23 peaked directly after surgery, decreased 6 h after surgery, and increased again 24 h after surgery (Figure 5). Potential variables of interest influencing the course of FGF-23 serum concentration was duration of surgery (*p* < 0.1, Table 4), tending to increase fluctuation.

#### 3.1.4 CIRBP Serum concentration

CIRBP was detectable at all investigated timepoints. Serum concentration increased postoperatively although not statistically relevant (Figure 3). In comparison to baseline, CIRBP showed an increase at T1 then returning to baseline levels over time (Figures 4, 5). In the longitudinal model we found a signal that duration of surgery had a significant influence on the course of CIRBP serum concentration (*p* < 0.1, Table 4). There was no significant sex related difference in CIRBP serum concentrations (Figure 6).

As CIRBP is known to be upregulated by mild to moderate hypothermia (28–34°C) (40, 42), we analyzed CIRBP serum concentrations in patients treated with hypothermia during CPB. However, we did not detect significant differences in CIRBP serum levels between patients undergoing normothermic, mild-moderate hypothermic, or moderate deep hypothermic CPB.

## 4 Discussion

As both systemic inflammatory reactions and CLS are frequent complications after cardiac surgery in children with CHD, early diagnosis and treatment of patients at risk remain challenges in postoperative clinical management. This feasibility study was conducted to evaluate customized multiplex magnetic bead panels for the analysis of proinflammatory cytokines, previously described biomarkers for vascular leakage, acute kidney injury, and CIRBP in pediatric patients undergoing cardiac surgery at our center. CIRBP has been previously described as an inflammatory mediator and its serum or plasma concentration has been analyzed in adults after septic or hemorrhagic shock as well as after cardiac surgery. To our knowledge this is the first study to analyze serum concentrations of CIRBP in children with CHD before, during, and after cardiac surgery.

Peripheral blood concentrations of CIRBP have so far been analyzed using western-blot analysis and Enzyme-linked Immunoabsorbant Assay. As far as we know, CIRBP has not been analyzed using a customized magnetic bead panel and furthermore has not been detected in a pediatric cohort. Merck/Millipore established the customized magnetic bead panel and validated the measurements for our feasibility study using our serum samples. Using this technique, we were able to detect CIRBP as well as the other biomarkers including cytokines (IL-1β, IL-6, IL-8, MCP-1, and IL-10), markers for endothelial dysfunction (SDC-1, TM, Ang-2, and VEGF), and FGF-23 as a marker for kidney injury in a minimal serum sample volume (25 µl) in all enrolled patients. As blood sample volume is an important restriction in pediatric clinical studies, the ability to detect multiple analytes in a minimal serum volume is an important goal. Especially as we plan to enroll patients of different ages including neonates, infants, and toddlers up to adolescents with CHD for a larger follow-up clinical trial, the successful quantification of all analyzed biomarkers using the customized magnetic panel is an important achievement of this feasibility study.

### 4.1 Cytokines

We analyzed pro-inflammatory cytokines IL-1β, IL-6, and IL-8 serum concentrations in our cohort. Both IL-6 and IL-8 levels were increased directly after operation and remained increased during all analyzed postoperative timepoints compared to baseline values (Figures 3, 4). Analysis of the course of biomarker serum concentrations over time using linear mixed-effects models, IL-6 remained upregulated during all postoperative timepoints, whereas IL-8 showed a peak at 6 h and decreased 24 h after surgery (Figure 5). We did not see a significant regulation of IL-1β during the analyzed timepoints compared to baseline levels (Figures 3, 4), however linear mixed-effects models showed IL-1β increasing directly after operation and returning to baseline levels over time (Figure 5). The regulation of both IL-6 and IL-8 concentrations after CPB are consistent with previous clinical studies analyzing cytokine concentrations in children and infants after cardiac surgery (61–67). *Allan* et al. analyzed various cytokine plasma concentration in infants (median age 37 days) undergoing cardiac surgery at similar timepoints up to 24 h after CPB. Although this study concentrated on one age group, they detected similar dynamics of cytokine regulation with both IL-6 and IL-8 being upregulated directly after cardiac surgery (61). Furthermore, we recently analyzed cold-shock proteins as well as cytokines in dry blood spot samples of 23 patients with CHD of varying ages and diagnoses (median age 19 years) undergoing CPB and hypothermia. Both IL-6 and IL-8 were significantly increased after CPB and stayed elevated up to 24 h after surgery (62). However, in our previously published patient cohort we also detected a significant increase in IL-1β 24 h after CPB (62), which we did not see in our feasibility study. However, postoperative regulation of IL-1β has been controversial in clinical studies so far. Whereas no significant regulation of IL-1β has been reported in neither infants nor children after CPB (61, 66), there have been reports that IL-1β levels are significantly elevated in direct response to CPB in infants (63). As the median patient age was significantly higher in our last cohort the differing secretion dynamics might be due to age difference. Nevertheless, larger patients' cohorts are needed to further investigate a possible age dependency.

Analysis with linear mixed-effects models showed time and female sex as potential variables of interests in IL-1β serum concentration course (Table 4). Furthermore, we detected a trend in increased IL-1β serum concentrations at all timepoints in female patients (Figure 6) However, as we conducted a hypothesis-generating, non-confirmatory clinical study these signals need to be evaluated in future studies.

Furthermore, we analyzed the chemokine MCP-1 as well as the anti-inflammatory cytokine IL-10. Analyzing the course of serum concentration over time using linear mixed-effects models suggests MCP-1 and IL-10 to have a similar regulation dynamic. Both cytokines were upregulated directly after cardiac surgery and decreased back to baseline levels over time (Figure 4). This observed regulation is consistent with the results of various clinical studies analyzing infants and children after CPB (61, 64–66). We previously described an increase in both MCP-1 and IL-10 directly after CPB and decrease to baseline levels 24 h after surgery (62). However, other studies focusing on infants after pediatric cardiac surgery report different regulation of IL-10. *Gu* et al. detected an increase of IL-10 concentration directly after CPB showing a peak at 12 h after surgery before decreasing (63). While *Trotter* et al. showed that IL-10 levels remained elevated up to 7 days after CPB (67). As we did not analyze cytokine concentrations 12 h after CPB, we might have missed a potential further increase of IL-10 in our patient cohort. Nevertheless, a possible age-dependency needs to be investigated further.

### 4.2 Biomarker for endothelial dysfunction

SDC-1, Ang-2, and TM serum concentrations increased after operation. Both SDC-1 and TM increased both directly as well as 6 h after operation, whereas Ang-2 was upregulated during all postoperative timepoints as compared to baseline (Figures 3, 4). *In vivo* studies have shown that ischemia/reperfusion-induced injury leads to glycocalyx shedding and a significant increase in SDC-1 release (68). Various clinical studies have reported that SDC-1 concentration increases after cardiac surgery in both adults and pediatric patients (14, 38, 39, 69). *Bruegger* et al. analyzed SDC-1 in infants undergoing CPB with both beating heart aortic clamping and deep hypothermic circulatory arrest. They report a significant upregulation of serum SDC-1 concentration directly after CPB with SDC-1 remaining upregulated until intensive care unit (ICU) admission (39). In adults undergoing coronary artery bypass grafting with or without the use of CPB, SCD-1 was significantly upregulated during surgery and normalized during the first 24 h after surgery (69). However, previous clinical studies analyzing SDC-1 after pediatric cardiac surgery analyzed SDC-1 levels up to 2 h after surgery (38, 39). To our knowledge, this is the first report of SDC-1 levels 24 h after CPB in children. Analysis of the course of biomarker serum concentrations reveals a differing dynamic of the analyzed biomarkers for endothelial dysfunction as SDC-1 peaks at 6 h after operation whereas both Ang-1 and TM concentrations increase during the analyzed postoperative timepoints (Figure 5).

Changes in TM concentration after pediatric cardiac surgery have not been studied so far. TM has been shown to be associated with poor outcome in both children and adults suffering from sepsis (18, 19). In septic adults, TM serum concentration correlated with the risk of development of disseminated intravascular coagulation, multiple organ failure, or death during ICU stay (18, 19). TM enables thrombin-mediated activation of protein C playing a part in coagulation, fibrinolysis, and inflammation (70, 71). As it is released upon inflammation and endothelial cell damage (72), it remains an interesting potential biomarker for SIRS and CLS and its' regulation after pediatric cardiac surgery warrants further investigation.

Whereas Ang-2 levels increased after CPB, we report a significant decrease of VEGF-A directly after CPB and a slight increase during later timepoints although not statistically significant (Figures 3, 4). *Giuliano* et al. analyzed both Ang-2 and VEGF-A plasma concentrations in children undergoing CPB (median age 5 months) at the same timepoints as in our study (prior to operation, 0, 6 and 24 h after CPB). Ang-2 was significantly elevated 6 h after CPB and remained upregulated until 24 h after CPB. VEGF-A concentrations, however, were not significantly regulated after CPB (35).

SDC-1 has been associated with severe acute kidney injury as well as prolonged duration of stay on the intensive care unit as well as hospital stay after pediatric CPB (38). Ang-2 concentration at 6 h after CPB has been reported to show a correlation with CPB time and surgery complexity as assessed via RACHS-1. Furthermore, Ang-2 concentration was associated with the duration of stay on the ICU (35). VEGF concentration has been shown to be elevated after CPB in neonates and was associated with postoperative capillary leak syndrome (36). Elevated preoperative as well as postoperative VEGF-A concentrations have been associated with cyanotic congenital heart disease (36, 37).

Analysis with linear mixed-effects models revealed age, female sex, and duration of surgery as potential variables to influence both SDC-1 and TM course of serum concentrations over time, whereas Ang-2 concentration seems to be influenced by age and duration of surgery (Table 4). To our knowledge this has not been reported so far, however our study cohort represents a small and heterogenous group both in relation to age and cardiac defect. Additionally, there was no neonatal patient included in our study cohort. Therefore, both associations with cardiac anomalies as well as age and adverse clinical outcome need to be investigated further.

### 4.3 Fibroblast growth factor (FGF-23)

FGF-23 has been associated with AKI after CPB in children (59, 60). FGF23 is an osteocyte-derived hormone playing an important role in phosphate and vitamin D homeostasis (73). Serum FGF23 has been shown to be increased in both early stages of chronic kidney disease (74) as well as in early stages of acute kidney injury (75, 76). Preoperative FGF23 serum concentration was associated with AKI development after CPB suggesting FGF23 as a suitable screening marker for AKI (60). Additionally, FGF23 has also been described to be associated with inflammatory processes in experimental studies (77). We report a significant increase at both directly as well as 6 h after surgery in our patient cohort (Figures 3, 4). AKI occurred in 5 patients (26%, 22% in female and 30% in male patients). In accordance with AKI, FGF-23 serum concentrations seem to be influenced by the duration of surgery (Table 4). We report that FGF23 serum concentrations could be measured adequately using our customized panel.

### 4.4 CIRBP

CIRBP has been reported as a key player in the innate inflammatory response. Both *in vitro* and *in vivo* studies have shown that CIRBP enhances inflammation by inducing proinflammatory cytokines and DAMPs release (44). CIRBP antiserum as well as blockage of its receptors have been shown to reduce systemic inflammation, organ dysfunction, and mortality in *in vivo* sepsis and hemorrhagic shock model (44, 47). We could detect CIRBP in all patients, with increasing serum concentration after cardiac surgery (Figures 3, 4). Furthermore, linear mixed-effects models revealed duration of surgery having a potential influence on CIRBP serum concentrations (Table 4). However, as the purpose of this feasibility study was to establish the validity of our customized magnetic bead panel in order to analyze multiple analytes using only a small sample volume, the question of the present study is related to the measurability of biomarkers in the setting of CPB in children. Due to the size of our cohort, the potential value and significance of CIRBP as a biomarker was not assessed in this study. Nevertheless, the stable course for 17 out of 19 patients could raise the question if for the 2 patients with higher initial values and different progression the post-surgery recovery was divergent from the rest of the group. This has to be investigated in a larger sample. Following the positive results of this feasibility study, we further conducted a larger trial including 108 patients and are in the process of analyzing the results. To our knowledge this is the first study analyzing CIRPB in a pediatric population. So far, CIRBP has been detected in peripheral blood of patients suffering from septic and hemorrhagic shock (44, 46) with high CIRBP concentrations correlating with a poor survival rate (46). Furthermore, CIRBP has been detected in adult patients after cardiac surgery with CPB showing a correlation between duration of CPB and postoperative lung dysfunction (78). Whereas serum CIRBP was detectable in all patients suffering from hemorrhagic shock with a mean blood collection time of 43 h after onset of shock, CIRBP could not be detected in the control group (44). *Zhou et al*. report elevated plasma levels of CIRBP in septic patients and suggests CIRBP as an independent predictor for sepsis mortality as non survivors showed significantly higher CIRBP levels compared to survivors (46). *Chen* et al. analyzed CIRBP plasma concentration using Enzyme-linked immunosorbent-Assay in 31 adult patients (median age 60 years; 17 male, 14 female) undergoing cardiovascular surgery at 1 day before surgery, as well as 6 h, and 1, 3, and 5 days after surgery. They observed a significant increase in serum CIRBP 6 h after CPB that returned to baseline levels 5 days after surgery. CIRBP plasma levels were upregulated up to 1,300pg/ml postoperatively. Furthermore, they also observed a correlation between increased CIRBP concentrations with increasing CPB duration (78).

Interestingly, hypothermia did not have an influence on CIRBP concentration in serum. We recently investigated the gene expression of both cold-shock proteins RNA-binding motif 3 (RBM3) and CIRBP in patients treated with targeted-temperature management (33°C for 24 h) after cardiac arrest. CIRBP mRNA expression showed a tendency of upregulation after 24 h of cooling and decreased significantly during the following 48 h, whereas RBM3 was significantly elevated after 24 h of cooling (79). *Chen* et al. also did not observe a significant regulation of CIRBP due to hypothermia during CPB (mean temperature during CPB 31.6 ± 1.4°C) (78). However, the number of patients treated with hypothermia in our study was small and varied in temperatures between 28 and 32.1°C. Therefore, potential effects due to hypothermic treatment might not have been detected. Furthermore, we did not analyze the effect of body temperature after CPB on CIRBP concentration. As both slow rewarming after hypothermia during CPB and hyperthermia/fever could occur after pediatric cardiac operation, the possible effect of body temperature following CPB on CIRBP serum concentration needs to be analyzed in larger patients cohorts. In the following clinical study, we plan on assessing temperature along with other vital parameters up to 72 h after cardiac operation to investigate possible correlations.

### 4.5 Sex-associated differences

It is known that there are sex dependent differences concerning the incidence of specific cardiac defects in CHD. Transposition of the great arteries and left-sided obstructions occur more often in males, whereas atrial septal defects and Ebstein's anomalies are reported to be more frequent in female patients (80). Moreover, retrospective analyses report that male patients are more likely to undergo complex high-risk cardiac surgery (81). This aspect might influence early postoperative morbidity and potentially biomarker concentration. However, data on sex associated differences regarding postoperative mortality is to date inconsistent (82, 83). *Trotter* et al. investigated sex related differences in postoperative cytokine levels in 18 infants and children after CPB. All patients showed significant increases in IL-8 and IL-10 after surgery, however IL-10 plasma concentration was reported significantly higher in female patients (67). We did not observe a sex dependent regulation in IL-10 concentrations, but we did observe a non-significant trend in higher IL-1β at all respective timepoints in male patients (Figure 6). To our knowledge, a correlation between gender and CIRBP expression and secretion has not been investigated so far. We did not detect sex related differences in CIRBP serum concentrations (Figure 6). *Trotter* et al. reported a significant increase in the sex steroid progesterone in both male and female patients after CPB, but observed that only male patients developed multiple organ dysfunction (67). Considering the relatively small number of patients in their study and differences in clinical outcome the reported sex-dependent differences might also be related to clinical outcome, age, or original clinical syndrome. (67), This needs to be considered in our feasibility study as well. Although, both male and female patients had a comparable risk for in-of hospital mortality as assessed by RACHS-1 and operation time as well as time on CPB was similar (Table 2), length of PICU stay and mean VIS during the first 24 h after surgery was higher in male patients (Table 3).

### Limitations

The present feasibility study was conducted as a single-center study enrolling a small number of consecutive heterogenous patients in regards to sex, age, cardiac anomalies, and subsequently complexity of cardiac surgeries. In all analyzed patients, surgery was conducted using CPB. The study protocol did not include a control group to evaluate the analyzed biomarkers in healthy children or children without cardiac surgery. Unfortunately, it would not have been justifiable from an ethics perspective, as we would need to draw blood from healthy infants and children, which can be a traumatic experience in pediatric patients. Since our cohort already has central venous access before the start of the operations, it was completely painless and easy to obtain blood samples at all time points from our pediatric patients. Furthermore, the concentration of analyzed biomarkers could be influenced by preoperative preparation of patients such as fasting or anesthesia induction as the baseline values were obtained after anesthesia induction directly before surgery. In summary, the results of this pilot study need to be confirmed by a clinical study analyzing a larger number of patients.

## 5 Conclusion

A valid measurement of the regulation of CIRBP in the clinical setting of cardiac surgery in children is possible. Measurements of potential biomarkers in a larger clinical trial in a pediatric patient cohort using small serum samples during cardiac surgery are essential to validate the promising preliminary results.

This feasibility study shows that multiple analytes can be quantified in a minimal serum volume (25 µl) using a customized multiplex magnetic bead panel. Moreover, a valid measurement of the release of CIRBP into the circulation in a clinical setting of cardiac surgery in children was possible. Measurements of potential biomarkers in a larger clinical trial in a pediatric patient cohort using small serum sample volumes collected during cardiac surgery are essential to validate these promising preliminary results.

## Data Availability

The original contributions presented in the study are included in the article/Supplementary Material, further inquiries can be directed to the corresponding author.
